# Involvement of Ahr Pathway in Toxicity of Aflatoxins and Other Mycotoxins

**DOI:** 10.3389/fmicb.2019.02347

**Published:** 2019-10-10

**Authors:** Francisco Arenas-Huertero, Montserrat Zaragoza-Ojeda, Juana Sánchez-Alarcón, Mirta Milić, Maja Šegvić Klarić, José M. Montiel-González, Rafael Valencia-Quintana

**Affiliations:** ^1^Experimental Pathology Research Laboratory, Children’s Hospital of Mexico Federico Gómez, Mexico, Mexico; ^2^Rafael Villalobos-Pietrini Laboratory of Genomic Toxicology and Environmental Chemistry, Faculty of Agrobiology, Autonomous University of Tlaxcala, Tlaxcala, Mexico; ^3^Mutagenesis Unit, Institute for Medical Research and Occupational Health, Zagreb, Croatia; ^4^Department of Microbiology, Faculty of Pharmacy and Biochemistry, University of Zagreb, Zagreb, Croatia

**Keywords:** *Aspergillus*, *Alternaria*, AFB1, alternariol (AOH), CYP1A1

## Abstract

The purpose of this review is to present information about the role of activation of aflatoxins and other mycotoxins, of the aryl hydrocarbon receptor (AhR) pathway. Aflatoxins and other mycotoxins are a diverse group of secondary metabolites that can be contaminants in a broad range of agricultural products and feeds. Some species of Aspergillus, Alternaria, Penicilium, and Fusarium are major producers of mycotoxins, some of which are toxic and carcinogenic. Several aflatoxins are planar molecules that can activate the AhR. AhR participates in the detoxification of several xenobiotic substances and activates phase I and phase II detoxification pathways. But it is important to recognize that AhR activation also affects differentiation, cell adhesion, proliferation, and immune response among others. Any examination of the effects of aflatoxins and other toxins that act as activators to AhR must consider the potential of the disruption of several cellular functions in order to extend the perception thus far about the toxic and carcinogenic effects of these toxins. There have been no Reviews of existing data between the relation of AhR and aflatoxins and this one attempts to give information precisely about this dichotomy.

## Introduction

Aflatoxins and other mycotoxin are activators of the aryl hydrocarbon receptor (AhR), are a structurally diverse group of fungal secondary metabolites and a toxigenically and chemically heterogeneous assemblage. They are ubiquitous contaminants in a broad range of agricultural commodities and feed (Table 1) and can contaminate the food supply at any time during production, processing, transport or storage (Bräse et al., 2009). Currently, far more than 400 mycotoxins are produced by some 350 fungi species (Kuhn and Ghannoum, 2003), but scientific attention is given mainly to those that have proven to be carcinogenic and/or toxic (Zain, 2011). The aflatoxins and other toxins that can act as activators to AhR can be acutely or chronically toxic, depending on the kind of toxin, the dose, health, age and nutritional status of the exposed individual or animal, and the possible synergistic effects between mycotoxins (Bennett and Klich, 2003). Their ingestion, inhalation, or skin absorption can cause diseases in humans and animals and their greater impact on human health in industrial countries is due to the chronic exposure (Gil-Serna et al., 2014). In this case, aflatoxins are associated with different biological effects like carcinogenic, mutagenic, teratogenic, estrogenic, hemorrhagic, immunotoxic, nephrotoxic, hepatotoxic, dermotoxic and neurotoxic ones, including displays of both tumor and antitumor effect, cytotoxic, and antimicrobial properties (see Table 1). Their impact in human diseases has been the subject of intensive study since the early 1960s, since aflatoxicosis and other mycotoxicosis can provoke tumor formation or even rapid death (Bräse et al., 2009).

**TABLE 1:** Toxic effects of several mycotoxins in human.

**Mycotoxin**	**General effects**	**Mycotoxin related symptoms/disease in humans**	**References**
Aflatoxins	Hepatotoxic; immunotoxic, genotoxic, mutagenic, teratogenic carcinogenic	Acute/chronic liver disease, (hepatocellular carcinoma, hemorrhagic necrosis, fatty acid infiltration in liver cells), lung cancer, child growth impairment, immunosuppression, Kwashiorkor disease, change in protein metabolism and micronutrients (Zn, Fe, vitamin A), vomiting, abdominal pain, anorexia, diarrhea, depression, jaundice, photosensitivity, pulmonary or cerebral edema, encephalopathy, pulmonary interstitial fibrosis, reproductive system effects (particularly male)	Steyn, 1995; Bryden, 2007; Bräse et al., 2009; Marin et al., 2013; Gil-Serna et al., 2014; Wu et al., 2014
*Alternaria* toxins	Genotoxic, mutagenic, teratogenic carcinogenic	*Onyalai* disease, cardiovascular collapse, gastrointestinal hemorrhage, lung, and esophageal cancer	Ostry, 2008; Siegel et al., 2010; Marin et al., 2013; Gil-Serna et al., 2014; Lee et al., 2015; Fan et al., 2016
Fumonisin B_1_	Neurotoxic, hepatotoxic, nephrotoxic, immunosuppressive, carcinogenic, teratogenic	Neural tube defects, esophageal and liver cancer, inhibit the uptake of folic acid via the folate receptor	Steyn, 1995; Ueno et al., 1997; Marasas, 2001; Missmer et al., 2006; Bryden, 2007; Voss et al., 2007; Bräse et al., 2009; Zain, 2011; Gil-Serna et al., 2014; Wu et al., 2014
Ochratoxin A	Nephrotoxic, hepatotoxic, immunotoxic, carcinogenic, genotoxic teratogenic	Renal diseases (Endemic nephropathy, urothelial, and kidney tumors)	Steyn, 1995; Schwartz, 2002; Bryden, 2007; Bräse et al., 2009; Gil-Serna et al., 2014; Wu et al., 2014

Some molds are capable of producing more than one toxin and some toxins are produced by more than one fungal species (Bräse et al., 2009; Zain, 2011). Aflatoxins are commonly found in many food products, such as cereals, fruits, nuts, and meat, among others.

## Interactions of Aflatoxins and Other Foodborne Mycotoxins With AhR

The major foodborne mycotoxins of public health interest are aflatoxins. However, there are several foodborne mycotoxins (*Alternaria* toxins, fumonisin B_1_, ochratoxin A, and patulin) that should not be excluded due to their broad toxicity, as well as ability to activate AhR-pathway, the focus of this review. These mycotoxins account annually for millions of dollars losses worldwide in human health, animal health and discarded agricultural products (Zain, 2011).

## Aflatoxins

**FIGURE 1:**
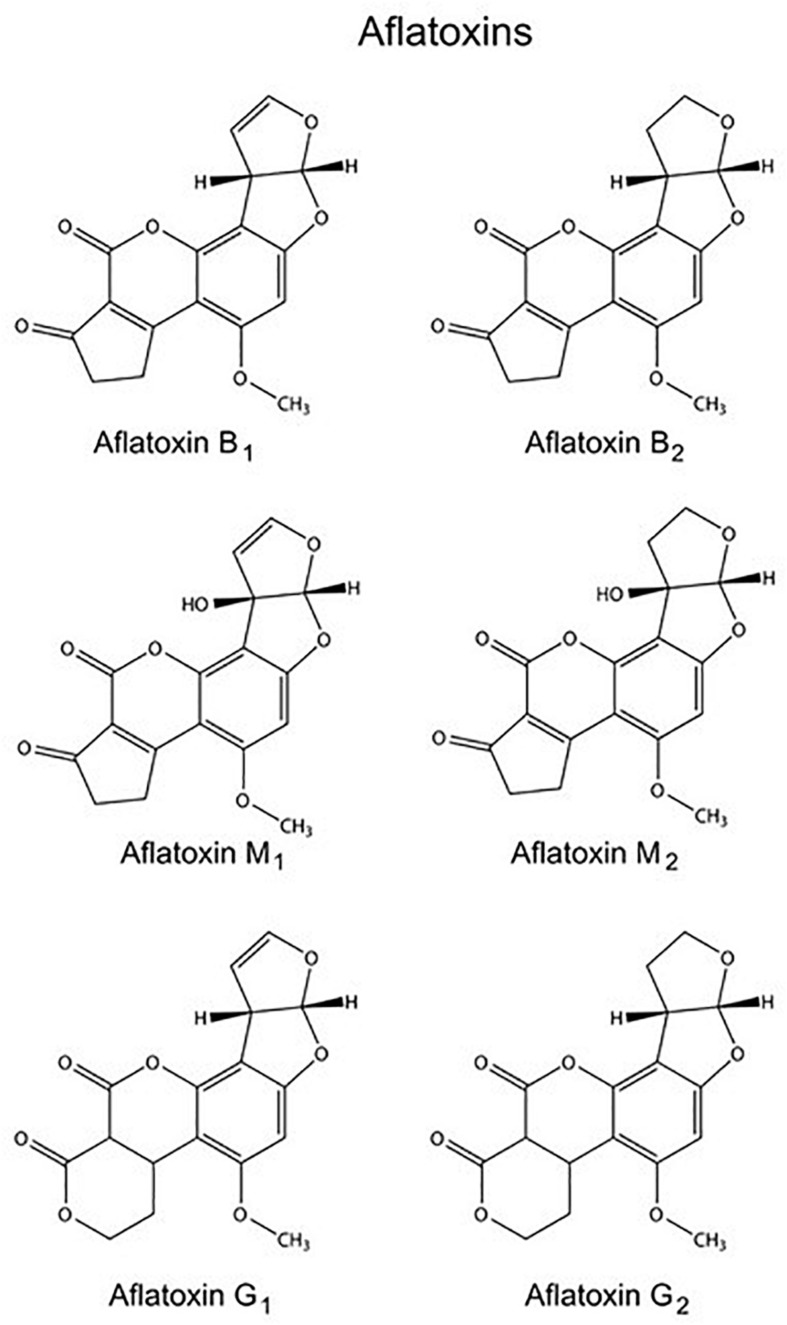
Molecular structures of several aflatoxins.

The name “aflatoxin” (*A. flavus* toxin) was assigned to a number of polyketide-derived furanocoumarins carcinogenic metabolites produced mainly by fungi *Aspergillus flavus* and *Aspergillus parasiticus* (Kensler et al., 2011). The aflatoxins were structurally identified in the early 1960s and over the last 50 years have been extensively studied with respect to their mechanism of action, including their mutagenic and carcinogenic activity (Valencia-Quintana et al., 2014). Collectively, aflatoxins are a group of approximately 20 chemically related metabolites, although the most important and naturally occurring are aflatoxins B_1_ (AFB_1_), B_2_, G_1_, and G_2_ (Figure 1). The AFB_1_ is in this group the most potent hepatotoxin with highly toxic and carcinogenic characteristics for many animal species (Hendricks, 1994).

Aflatoxins are metabolically activated in the liver by major CYP isoenzymes as CYP1A1, CYP1A2, and CYP3A4, their biotransformation produces a highly reactive AFB_1_-8,9-exo-epoxide and 8,9-endo-epoxide that can form adducts with DNA and RNA. In addition, CYP1A1 which also participates in AFB_1_ transformation into epoxide, represents the highest fraction of extrahepatic CYP. After binding to DNA, the predominant 8,9-dihydro-8-(N7-guanyl)-9-hydroxy AFB_1_ (AFB_1_-N7-Gua) adduct is formed. AFB_1_-N7-Gua may be converted to two secondary lesions, the one with apurinic site and the other with a more stable ring opened AFB_1_-formamidopyrimidine (AFB_1_-FAPY) adduct; the latter far more persistent *in vivo* than AFB_1_-N7-Gua. Sufficient evidence from animal and epidemiological studies, such as the demonstration of a specific mutation in the *TP53* gene, led to the AFB_1_ classification as a human carcinogen by the International Agency for Research on Cancer (IARC) (Hendricks, 1994; IARC, 2002; Krska et al., 2008; Valencia-Quintana et al., 2014). Among mycotoxicosis described in humans, aflatoxicosis is of the greatest concern; repetitive aflatoxin outbreaks were reported in recent years in Kenya, India, and Malaysia, often accompanied by fumonisins (Shephard, 2004; Lewis et al., 2005). In acute mycotoxication, it can cause impaired growth in children, vomiting, abdominal pain, pulmonary edema and liver necrosis (Valencia-Quintana et al., 2012).

Because of its planar structure, it could act as activators for the AhR, at the moment there is not evidence to prove that any mycotoxin is an agonist of AhR. That was first seen in H4IIE cells; AFB_1_ induced an increase in CYP1A activity and CYP1A transcription, which was associated with an enhanced AhR activity, suggesting AhR pathway activation as a toxicity mechanism of AFB_1_ (Mary et al., 2015). The major human cytochrome P450 (CYP) enzymes involved in aflatoxin metabolism are CYP1A1, CYP3A4, 3A5, 3A7, and 1A2 (Tan et al., 2004; Marin et al., 2013). The CYP enzymes activation is an indirect evidence of activating AhR pathway. It is known that AFB_1_ causes carcinogenic, mutagenic and teratogenicity effects (Krska et al., 2008; Zhang et al., 2015), as well as immunotoxicity, hepatotoxicity and even death in farm animals and humans (Table 1). The AFB_1_ causes micronucleus (MN), sister chromatid exchanges (SCE), unscheduled DNA synthesis, and chromosomal strand breaks as well as adducts in rodent and human cells (Turkez and Geyikoglu, 2010). The mechanism of AFB_1_ cellular damage has not been completely elucidated. Reactive oxygen species (ROS) and lipid peroxidation (LPO) have been considered to be an important mechanisms in the toxicity. The ROS and LPO are created via AhR activation and its target genes as several CYPs, already mentioned above. In the following sections, this review will describe how the AhR pathway participates via cytochrome activation and the ROS production.

### Alternaria Toxins

**FIGURE 2:**
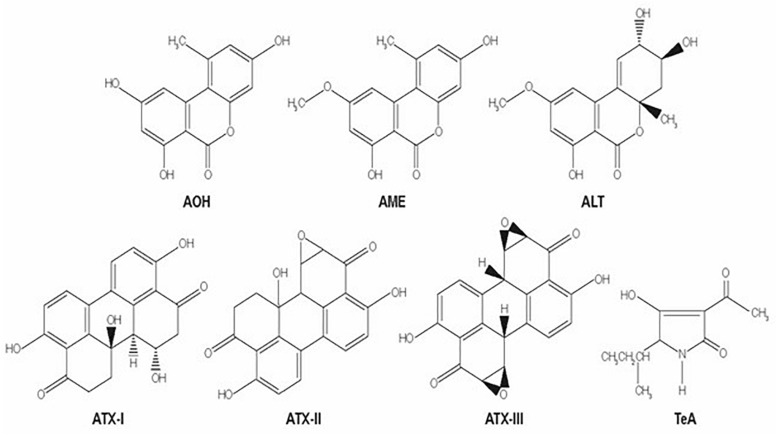
Molecular structures of several *Alternaria* mycotoxins: alternariol (AOH), alternariol monomethyl ether (AME), altenuene (ALT); the perylene derivatives altertoxins (ATX-I, -II, -III); and the tetramic acid derivatives, tenuazonic acid (TeA).

The *Alternaria* genus is able to produce different mycotoxins, *A. alternata* is considered to be the main mycotoxin producer. *Alternaria* mycotoxins include a small percentage of more than 70 phytotoxins, and only few have been physicochemically characterized (Dall’Asta et al., 2014). They belong to three different structural groups: the dibenzopyrone derivatives, alternariol (AOH), alternariol monomethyl ether (AME), and altenuene (ALT); the perylene derivatives altertoxins (ATX-I, -II, -III); and the tetramic acid derivatives, tenuazonic acid (TeA) and iso-tenuazonic acid (iso-TeA) (Schrader et al., 2001; Ostry, 2008). The chemical structures of these mycotoxins are presented in Figure 2 (Zain, 2011).

Alternariol monomethyl ether (AME) and alternariol (AOH) are not very toxic, but exert mutagenic, genotoxic and estrogenic activity (Schrader et al., 2001; Fehr et al., 2009; Dall’Asta et al., 2014), and also inhibit topoisomerase I and II (Fehr et al., 2009; Schreck et al., 2012; Dall’Asta et al., 2014). These toxins are planar benzopyrones that are quite similar to benzo(a)pyrene [B[*a*]P], a polycyclic aromatic hydrocarbon, and are metabolized by CYP1A1 and 1A2 cytochromes, which generate epoxides with marked activity on DNA and protein (Schreck et al., 2012). AOH and AME induce DNA strand breaks in cell lines (Pfeiffer et al., 2007), unscheduled DNA synthesis in cultured human amnion FL cells, chromosomal aberrations and sisters chromatid exchange in human peripheral blood lymphocytes, mutation in V79 cells and transformation of NIH 3T3 cells (Brugger et al., 2006; Schobert and Schlenk, 2008; Pahlke et al., 2016). These two toxins are AhR activators in mouse hepatoma cells (Schobert and Schlenk, 2008; Schreck et al., 2012; Pahlke et al., 2016). At very low concentrations altertoxin-II induces CYP1A1, which results in the production of ROS (Pahlke et al., 2016) that can cause DNA damage. However, DNA damage does occur, likely due to the generation of direct DNA adducts or the ROS induction, independently of cytochrome activity (Schreck et al., 2012; Pahlke et al., 2016). TeA is considered to be the most toxic among the *Alternaria* mycotoxins. It inhibits protein synthesis and is biologically active, exerting cytotoxic, phytotoxic, antitumor, antiviral and antibiotic effects (Schobert and Schlenk, 2008; Siegel et al., 2010), and has been deemed responsible for the outbreak of “onyalai” disease (Siegel et al., 2010). Also *Alternaria* toxins in grains might be responsible for esophageal cancer and other diseases (Dall’Asta et al., 2014; Table 1). Still, little is known about the mechanisms of action of *Alternaria* toxins.

## Fumonisin B_1_

Since their discovery in 1988 (Gelderblom et al., 1988), fumonisins have been the subject of numerous toxicological investigations (Voss et al., 2007). Fumonisins are a group of mycotoxins with a strong structural similarity to sphinganine, the backbone precursor of sphingolipids, and are produced primarily by *Fusarium* species commonly associated mainly with cereal grains *Fusarium verticillioides*, *F. proliferatum* strains (García and Heredia, 2006; Marin et al., 2013). These toxins have been epidemiologically and experimentally associated with human esophageal cancer (Cortez-Rocha et al., 2003) and birth defects due to interference with cellular folate uptake (Stevens and Tang, 1997; Voss et al., 2007). FB_1_ is a cancer promoter, but a poor cancer initiator; based on toxicological evidence, the IARC has classified FB_1_ as a possible human carcinogen (group 2B) (IARC, 2002). FB_1_ co-occurred with AFB_1_ in a high-incidence area in human primary hepatocellular carcinoma in China, suggesting that the mixture may be involved in the development of the disease (Li et al., 2001). Toxic properties in general and the symptoms related to human exposure to FB_1_ are listed in Table 1.

**FIGURE 3:**
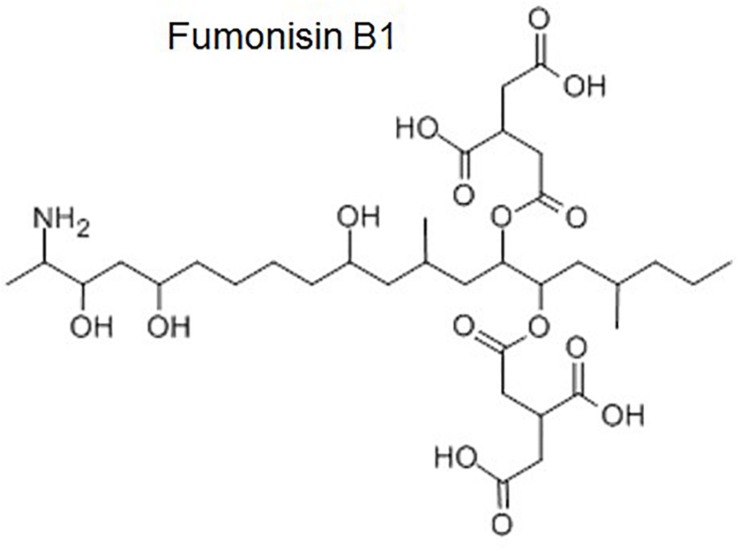
Molecular structure of fumonisin B1.

From a toxicological perspective, fumonisin B_1_ (FB_1_) is the most important fumonisin (Figure 3). The chemical name of this mycotoxin is 1,2,3-propanetricarboxylic acid, 1,10-(1-(12- amino-4,9,11-trihydroxy-2-methyltridecyl)-2-(1-methylpentyl)-1,2-ethanediyl)ester (European Food Safety Authority [EFSA], 2005), and it elicits a spectrum of toxicities likely mediated through mechanisms involving disruption of sphingolipid metabolism and sphingolipid-mediated processes (Voss et al., 2007). This mycotoxin inhibits ceramide (CER) synthase that catalyzes the acylation of sphinganine (Sa) and recycling of sphingosine (So), increases intracellular Sa and other cytotoxic sphingoid compounds. This imbalance is mainly responsible for the toxicity (Voss et al., 2007). Thus, alterations of Sa to So ratio in tissues, urine and blood have been proposed as potential biomarkers of FB exposure, but studies have not allowed an accurate validation (Solfrizzo et al., 2011). Also, the AhR activation was demonstrated when FB_1_ was evaluated in H4IIE hepatoma cells. As a result, this activation initiated the CYP1A1 and CYP1A2 activity (Mary et al., 2015).

### Ochratoxin A

**FIGURE 4:**
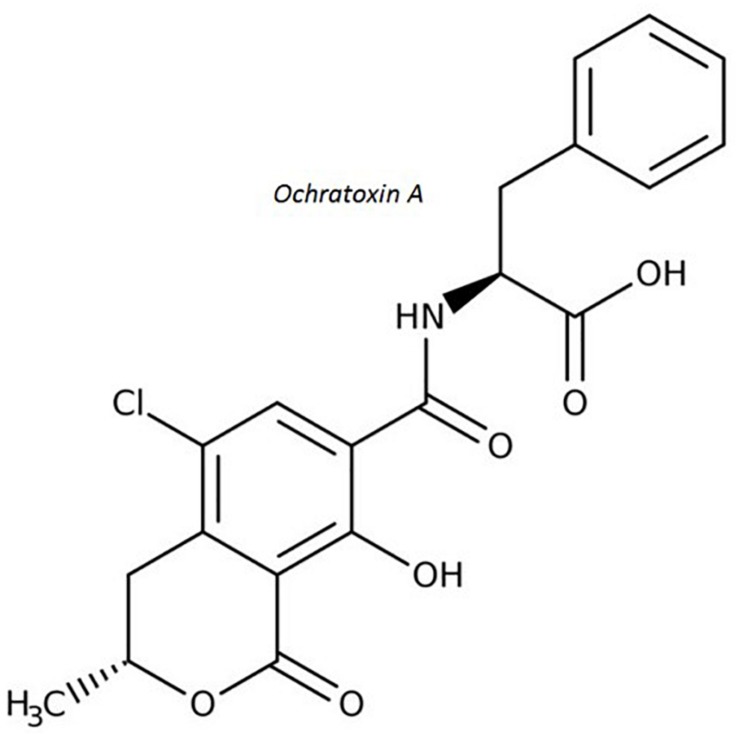
Molecular structure of ochratoxin A.

Ochratoxin A (OTA) is a secondary metabolite produced by *Penicillium verrucosum*, many species of the *Aspergillus* section *Circumdati* (*A. ochraceus*, *A. steynii*, and *A. westerdijkiae* are most important regarding OTA contamination in agricultural products) and a few *Aspergilli* section *Nigri* (Samson et al., 2007; Visagie et al., 2014). OTA (Figure 4) is recognized as a potential human health hazard (Table 1) associated with kidney disease in humans (García and Heredia, 2006) and has been classified as a possible human carcinogen (Tangni et al., 2002). Structurally, it is a phenylalanyl derivative of a substituted isocoumarin(R)-N-(5-chloro-3,4-dihydro-8-hydroxy-3-methyl-1-oxo-1H-2-benzopyran-7-y1)-carbonyl)-L-phenylalanine (Marin et al., 2013). Several mechanisms of OTA toxicity have been proposed including DNA damage coupled with forming direct DNA adducts via quinone formation and a network of interacting epigenetic mechanisms including inhibition of protein synthesis, oxidative stress, mitosis disruption and activation of specific signaling pathways (Mally, 2012; Pfohl-Leszkowicz and Manderville, 2012; Vettorazzi et al., 2013). In primary human hepatocytes cultures, it induced an increase in the transcription of the AhR gene and, thus, also CYP1A1 and 1A2 cytochromes gene transcription (Ayed-Boussema et al., 2012b).

### Patulin

**FIGURE 5:**
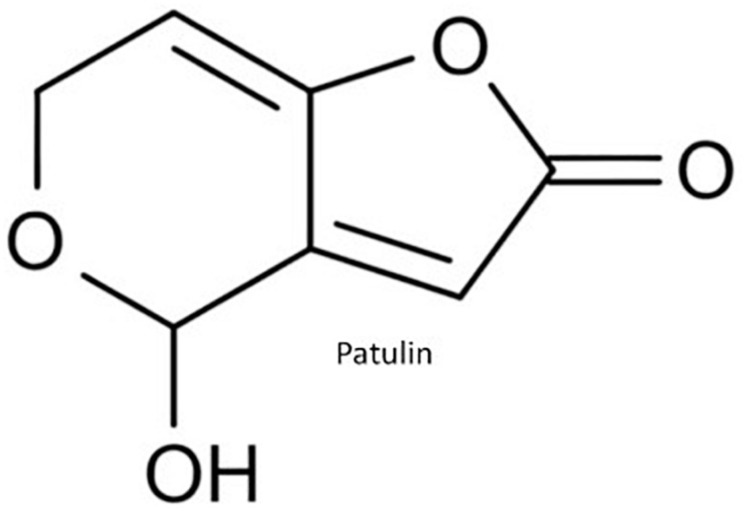
Molecular structure of patulin.

Patulin (PAT) (4-38 hidroxi-4H-furo(3,2-c)piran-2(6H)-ona) is mainly produced by *Penicillium expansum*, but other Penicillia can produce it including *P. crustosum, P. patulum* as well as *Aspergillus clavatus* (Samson et al., 2009; Figure 5). Apples, apple-based products and other fruits are major commodities contaminated with PAT and are responsible for PAT human exposure (Piqué et al., 2013). Animal model studies obtained convulsions, agitation, ulceration, edema, intestinal inflammation, vomiting and DNA damage in brain, liver and kidneys upon PAT exposure (McKinley and Carlton, 1980; de Melo et al., 2012). Chronic toxicity is related to neurotoxic, immunotoxic, genotoxic, and teratogenic effects in rodents (Boon et al., 2009). However, based on a lack of toxicological human data, the IARC has classified PAT in Group 3 as a non-human carcinogen (IARC, 2002).

PAT biotransformation has been poorly investigated; increase in CYP P450 content was reported in male mice and no increase was seen in the metabolic activity in human cells previously immortalized and transfected with human CYP 450 genes (Siraj et al., 1980; Lewis et al., 1999). The study that investigated involvement of AhR pathway in PAT toxicity is discussed later on in this review (section Activation of AhR Pathway by Other Mycotoxins).

## The Role of the Aryl Hydrocarbon Receptor: Historical Aspects

The study of the AhR reveals a two-dimensional history, each with its own characteristics. One is a reality of our time; namely, exposure to synthetic organic compounds and its consequences. Back at the beginning, in the 1970s, many toxicologists and, later, biochemists and molecular biologists, centered their attention on the toxicological implications of exposure to the halogenated polycyclic hydrocarbon 2,3,7,8-tetrachlorodibenzo-p-dioxin (TCDD) which was identified as a contaminant in the process of producing 2,4,5-trichlorophenol, a herbicide to which the workers who participated in its synthesis were exposed to (Schultz, 1957). After exposure, those workers suffered chloracne and *porphyria cutanea tarda* (Kimmig and Schulz, 1957). Later, studies proved that TCDD can elevate the expression of the δ- aminolevulinate synthase enzyme that participates in the heme biosynthesis (Poland and Glover, 1973). The second aspect is a rather serendipitous finding that appeared for reasons unknown at that time. In the early 1950s, studies observed that treating rats with the carcinogen 3-methylcholanthrene inhibited the hepatocarcinogenicity of 3′-methyl-4-dimethylamino azobenzene (Richardson et al., 1952). Afterward, it was proven that this carcinogenicity-inhibiting activity can be induced as well by other polycyclic aromatic hydrocarbons (PAH), such as B[*a*]P. Thus, it was demonstrated that these PAH can block the activity of an enzyme that modifies carcinogens, and it was suggested that, due to the constant use of B[*a*]P, it should be called B[a]P hydroxylase (Conney et al., 1957), which today corresponds to CYP1A1 hepatic cytochrome. Back then, in the 1960s, it was determined that the enzyme B[*a*]P hydroxylase, renamed at that moment as aryl hydrocarbon hydroxylase, or AHH, was inducible in some, but not all, syngeneic strains of mice (Nebert and Bausserman, 1970; Nebert et al., 1982). This response suggested the existence of a gene and AHH locus, denominated *Ah*, which is expressed, together with the enzyme, in the C57BL/6 strain, and corresponded to the *Ah*^*b*^ allele. Mice of the DBA/2 strain were not responsive, and that corresponded to the *Ah*^*d*^ allele (Nebert et al., 1972, 1982). Later, the role of the *Ah* locus was demonstrated in regulating the carcinogenicity, mutagenicity, teratogenicity and toxic responses to the PAH, based on inducing AHH activity (Nebert, 1986).

This provided the opportunity to test the inductive strength of AHH for TCDD and 3-methylcholanthrene, and subsequent research demonstrated that TCDD was 30,000 times more potent in AHH enzyme induction (Poland and Glover, 1974). As a result, this became the ideal test molecule for measuring all metabolisms of xenobiotic compounds in that period. Thanks to this, strains with phenotype *Ah*^*d*^ to 3-methylcholanthrene could be made responsive to TCDD (Poland and Glover, 1974). At that time, the study of steroid receptors was also in its apogee and researchers pondered the existence of a “receptor” with smaller affinity for 3-methylcholanthrene and a greater TCDD affinity, from the *Ah* locus that induces AHH (Yueh et al., 2003). Once the first radioactively-marked TCDD was synthetized, in 1976, the existence of a fraction bonded to the cytoplasm and another to the nucleus was successfully demonstrated by the differential fractioning method. Similarly to what occurred with the steroid receptors, once the TCDD bonded to its receptor, it was translocated to the nucleus with its *Ah* receptor (Poland et al., 1976). The weight of the receptor varied if it was isolated from the cytoplasm and, in fact, was heavier than that found in the nucleus (Gasiewicz and Bauman, 1987). This raised suspicions of the possible presence of other protein compounds. It was then demonstrated that induction of AHH activity requires the formation of the TCDD-AHR compound (Jones et al., 1984). In the 1980s, the nucleotide sequence to which the TCDD-AHR compound bonded on the AHH gene was identified, and it was confirmed that it corresponded to a gene of the group of enzymes denominated cytochromes, in this case *Cyp1a1*. This sequence was named the dioxin response element (DRE) (Jones et al., 1985), which was shown to have the ability to activate expression of heterologous genes. At first, its existence was demonstrated in mice, but later in rats and humans as well. In another part of the world, in Japan, studies were being conducted, not regarding the response to TCDD, but to other xenobiotic compounds, and those reports found the same sequence where the TCDD-AHR compound should bond, though they called it the xenobiotic response element (XRE) (Fujisawa-Sehara et al., 1988), this sequence is: 5′-TNGCGTG-3′ (Pohjanvirta, 2011). Biochemical evidences continue to reveal differences between the molecular form of AhR found in the cytoplasm (i.e., heavier) and the one existing in the nucleus. This latter finding suggested the presence of another, very similar, weight unit. This led to the clonation and determination of the AhR nuclear receptor translocator (ARNT), a protein that forms the complex with the receptor and generates the bond with TCDD (Hoffman et al., 1991). First, the cloning of the AhR gene in humans in 1994 made it possible to prove that the initial enzyme, AHH, corresponded to the cytochrome CYP1A1. After that, researchers undertook explorations of its expression in a large number of tissues as well as in other organisms (Pohjanvirta, 2011). This information then made it possible to look for its expression in different pathologies, such as those characterized by high *Cyp1a1* activity, which suggested a “cross-talk” in different pathways that run from the one in charge of metabolizing and eliminating toxic xenobiotic compounds to the alteration of such processes as the response to steroid hormones in organs like the ovaries and mammary glands (Kociba et al., 1978). From that moment forward, expression of *Cyp1a1* and *Cyp1b1* in response to the application of natural compounds, drugs, other PAHs, and other compounds in general such as mycotoxins, could indirectly reflect the activation and/or participation of the AhR pathway, as shown below, in a wide variety of processes, including proliferation, death/apoptosis, differentiation, cellular adhesion, and drug-expulsing proteins (Pohjanvirta, 2011).

Finally, we now know that this molecule that emerged initially from the response to toxic compounds, providing on this way a linear explanation of toxicology, specific “only” to this response, is actually a protein that is a master regulator situated above all general cellular processes, including proliferation, differentiation, cellular adhesion, death and others that will surely be described in the years to come.

## General Characteristics of the AhR Receptor

### Organization of the Gene

In order to know and understand better the gene function, it is important to focus the analysis on the sequence organization, from the promoter zone to the codifying region. Before beginning to describe its important molecular and sequential characteristics, it is vital to mention that the AhR gene provides a clear example of the evolution of proteins in organisms. Genes homologous to this receptor exist, from *Caenorhabditis elegans*, called AHR-1, which also possesses a protein that forms a pair with AHR-1 and AHA-1, which is the homolog of ARNT. Also, other organisms, like *Drosophila melanogaster*, have a *Spineless* that is homologous to human AhR; and *Tango* which is homologous to human ARNT. Gene conservation during evolution in pluricellular organisms has an element in common related to the differentiation processes in the early stages of development (Pohjanvirta, 2011). Detailed information related to what occurs in humans is presented below.

Thanks to the cloning of the close promoter region (Eguchi et al., 1994), it is important to mention that one of the principle characteristics is that this promoter is without a TATA box, which characterizes the promoters of specific tissue genes, and has three *cis* GC sites that act as bonding sites to the factor *trans* Sp1; a feature of gene promoters that have a constitutive expression. It also possesses a *cis* bonding site to CRE factors, and another E-box type site for binding to Myc. This analysis is a product of the study of the sequence -468 to -911 close to the promoter. The human gene promoter is longer than the mouse one and therefore generates a longer complete gene (promoter plus codifying region) of 6.6 pb vs. 5.5 pb, respectively. Harper et al. (2006) analyzed the presence of these *cis* binding sites to other transcription factors, at -5000 pb over the promoter sequence. Their results showed multiple *cis* sites to the factor *trans* HNF (hepatic nuclear factor) that numbered approximately 23 (Kaestner et al., 1994). There were also 2 *cis* DLX3 regions (Distal-less 3), to which this home box factor binds and functions as a placenta-specific transcriptional regulator (Morasso et al., 1999). In addition, it contains 11 BRN3-like sites that serve to bind to transcription factors of the POU family specifically expressed in the nervous system of mammals in development and in adult state (Korkalainen et al., 2005). Moreover, this complex participates in the differentiation and survival of motor and sensory neurons (McEvilly et al., 1996). There are also four *cis* sites to STAT6 (a signal transducer and transcription 6 activator) which belong to the family of the transcription factors that respond to cytokines such as IL-4, and to growth factors like TGF-β1 (Tanaka et al., 2005). In addition, there are 7 *cis* regions that bind to factors such as Tcf/Lef, other transcription factors that realize their nuclear response to Wnt signaling by interacting with β-catenin (Eastman and Grosschedl, 1999). Finally, there are 11 *cis* binding sites to potential nuclear receptors of the progesterone-, androgen-, glucocorticoid-, proliferation-activated peroxisome-, the farsenoid X-, and vitamin D- receptors (Harper et al., 2006). This full information on the *cis* sites inside the complete promoter makes it possible to see and understand the constitutive expression and specific tissue of the receptor, while also allowing us to comprehend one additional characteristic: the fact that it is a constitutive gene with ubiquitous expression; due to the important biological functions it fulfills (Hahn, 2002). It is especially critical during embryogenesis and fetal development in the first post-natal stages, after which its levels decrease significantly. This suggests that its role is required less in the final phases of an organism’s growth (Harper et al., 2006) and also allows for the explanation of the broad responses observed in several tissues and organs where xenobiotic compounds such as the aflatoxins, exert their hepato-, nefro- and neurotoxic activity as well as mutagenic and carcinogenic properties.

The open reading frame has 12 exons, which are organized and formed in the mature messenger and the important domains of the protein, as it will be described below.

### Important Domains of the Protein

The detailed information regarding the domains present in the protein was obtained principally by cloning mouse genes. The mouse’s peptic sequence revealed the presence of 805 residues of amino acids organized in domains present in those bHLH-PAS transcription factors (Burbach et al., 1992). These factors contain a binding region to DNA basic Helix-Loop-Helix (bHLH) and a pair of domains that have been found in the Per proteins (a factor that participates in regulating the circadian cycle), in ARNT (i.e., the protein with which AhR realizes heterodimerization) and, finally, with a protein (that participates in neuronal development in *Drosophila*) called “single-minded,” or Sim, which gives this domain its name: PAS (Pohjanvirta, 2011). Analyses based on the terminal amino domain show a region of basic amino acids residues, followed by the hHLH motif. The basic residues region is important for the AhR interaction with the response element *cis* sequence to the aryl hydrocarbons, ARE; while the hHLH motif is important for the realization of heterodimerization between AhR and Arnt. Ahead of this hHLH motif there are 2 repeated inversions regions of 51 amino acids residues that are also found in the PAS factors described above. These motifs perform multiple functions, such as directing the AhR union with 2 Hsp90 molecules and with the protein AIP (AhR interacting protein), or XAP2 (X-associated protein 2) in the cytoplasm (Ma and Whitlock, 1997), the bond to the ligand and the interaction of AhR and Arnt in the nucleus (Figure 2). The carboxy terminal contains three domains for the transcription activation (AT) that contain residues of amino acids that are acidic and rich in serine/threonine and glutamate. Between the PAS B and AT domains there is a small sequence of approximately 81 residues of amino acids that exerts an inhibiting activity on AT and so has been named the inhibiting domain (ID) (Ma et al., 1995).

### Associated Proteins and Formation of the AhR Complex

**FIGURE 6:**
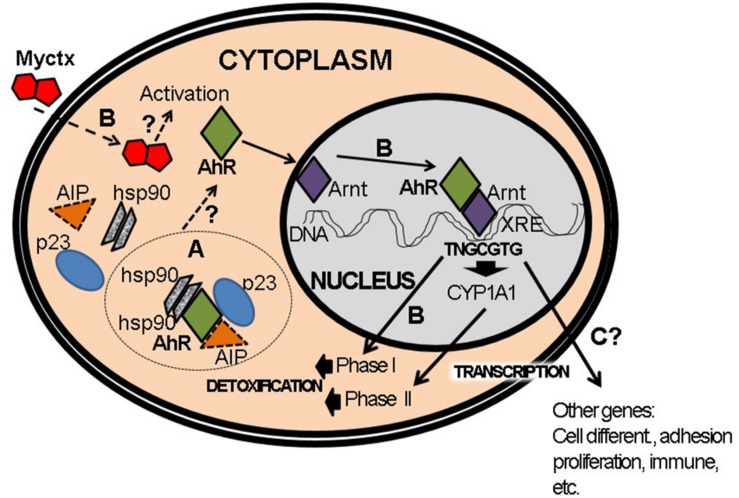
Activation of AhR pathway by mycotoxins (Myctx). (A) The AhR is a protein that interacts with hsp90, AIP, and p23 proteins in cytoplasm. (B) Under activation by Myctx, the transcription of phase I and phase II genes is started in order to metabolize and detoxify. It is not proved if the Myctx are ligands/agonists of AhR, which is indicated by a question mark (?). (C) This activation can also start transcription of several genes such as those involved in cell differentiation, adhesion, proliferation and immune response, but in the case of Myctx these have to be proved if it is performed as ligands/agonists (?).

One must recall that, historically, the study of the AhR was always conducted based on the results observed during exposure to, and interaction with, TCDD. The AhR in the cytosolic fraction was shown by ultracentrifugation to have a 9S sedimentation value. Upon adding the ligand, i.e., TCDD– this value decreases to 6S and, moreover, is found in the nuclear fraction (Pohjanvirta, 2011). These differences revealed the existence of two different forms in the two cellular compartments. In addition, electrophoretic studies demonstrated that this weight difference was due to the fact that the receptor protein in the cytoplasm is found in complex tetrameric compounds by that same receptor and three proteins with molecular weights of 96, 88, and 37 kDa, the first two corresponding to two isoforms of mouse HSP90 (Chen and Perdew, 1994). The protein XAP2 (X-associated protein 2) (Meyer et al., 1998), as well as AIP (AhR-interacting protein) (Ma and Whitlock, 1997) or ARA9 (AhR-associated protein 9) (Carver et al., 1998) is the one that corresponds to molecular weight 43–37 kDa (Figure 6). It has been shown that the interaction between Hsp90 and the AhR takes place in the PAS-B motif, and that this interaction is required to create the conformational change that allows binding with the ligand. The protein AIP has three repeated sequences of tetratricopeptides that allow protein-protein interaction (Schreiber, 1991). This explains why the receptor protein in the cytoplasm is more stable and has a half-life of approximately 28 h. This data corresponds to the earlier description which indicated that the onset of the induction of CYP1A1 does not require protein synthesis. After treatment with a ligand (TCDD or B[*a*]P), the receptor’s half-life is reduced to just 3 h (Ma and Baldwin, 2000). Afterward, the AhR protein suffers degradation by the 26S proteasome (Pohjanvirta, 2011), which takes place in the nucleus (Figure 6), an important site for the degradation of other transcription factors in addition to AhR (Roberts and Whitelaw, 1999), such as TGF-β1 (Nebert, 1986; Lo and Massagué, 1999) and MyoD (Floyd et al., 2001).

## The Canonical Pathway of AhR Activation Through Interaction With Arnt

In order to explain the canonical pathway of AhR activation (Figure 2), the description must be based strongly on the activation of the genes studied that participate in the detoxification mechanism, or in phase I and phase II of the xenobiotic compounds metabolism such as the cytochromes *Cyp1a1* and *Cyp1a2*. This history starts with the binding of the ligand to the AhR, that binds and causes a conformational change in the PAS A domain which enables demonstration from the residues of amino acids 55–75 to a nuclear localization signal, allowing its translocation to the nucleus (Figure 2). As mentioned previously, it dissociates in the cytoplasm of the complex of 2 molecules of Hsp90, and translocates to the nucleus in a process fostered by importins (Ikuta et al., 1998). Once inside the nucleus, AhR is dimerized with another protein from the same family as factors bHLH-PAS, denominated Arnt. Arnt is only localized in the nucleus and this protein is also organized in domains similar to those that contain AhR, i.e., toward the amino terminal end we find the bHLH domain and the nuclear localization signal, followed by the PAS A and PAS B domains (Figure 2). Finally, the activation domain is contained toward the carboxyl terminal end (Pohjanvirta, 2011). The dimerization between AhR and Arnt is realized through the HLH regions (Reisz-Porszasz et al., 1994; Fukunaga et al., 1995) and is stabilized by a conformational change in the PAS A region (Soshilov and Denison, 2008). Observations have shown that modifications by phosphorylation occur for the binding function to the DNA via the protein kinase C (Mahon and Gasiewicz, 1995). Binding to the ARE *cis* site in the promoter sequences takes place with the consensus sequence 5′-TNGCGTG-3′, which is seen in many genes, such as cytochrome CYP1A1, which contains 8; CYP1A2, which contains 1, and CYP1B1, which contains 3 sequences (Pohjanvirta, 2011). For this reason, it is said that these genes are regulated by the canonical pathway and that CYP1A1 is the model gene for the study of the response to AhR. In the context of the binding, AhR binds to the middle of the 5′-T/NGC site, while Arnt binds by occupying the second half of 5′GTG (Bacsi et al., 1995). We also know that a series of nucleotides located near the ARE are important because its *cis* element contains the optimal sequence: GGGTGNAT(C/T)GCGTGACNNCC. This sequence establishes contact with the bHLH domain of the AHR/ARNT heterodimer (Chapman-Smith and Whitelaw, 2006). We also know that the promoter has a second sequence or response element to AHR II, whose sequence is CATG(N)C(T/A)TG, and which is conserved in humans, rats and mice, but does not seem to influence the canonical response to ARE’s action on the *cis* site (Pohjanvirta, 2011). Thanks to the exhaustive studies that have been conducted on the regulation of the expression of the cytochromes during AhR activation, it was possible to identify various genes that have AREs (Gasiewicz et al., 2008), which can be grouped as follows:

1. Phase I genes of the metabolism of xenobiotics: CYP1A1, CYP1A2, CYP1B1, CYP2A5, CYP2S1, and CYP4B1.
2. Phase II genes of the metabolism of xenobiotics: aldehyde dehydrogenase 3A1, glutation-S-transferase, NAD(P)H- quinone oxidoreductase-1, UDP glucuronosyltransferase 1A1, and 1A6.
3. Cell cycle suppressors: p21, p27.
4. Cell cycle activators: c-jun, s-myc, junD, Insulin-like growth factor binding protein-1.
5. Others: Bax, cathepsin D, cyclooxygenase -2, epiregulin, filaggrin, Slug, etc.

There is also one exceptional case; that of poly/ADP-ribose polymerase, which contains 16 XRE *cis* sequences (Ma, 2002). One aspect of the consequences of cytochrome activation is the generation of modifications of the xenobiotic compounds that induce their activation and achieve control of the receptor activation while reducing ligand concentrations in the cell (Gu et al., 2000). All currently available evidence demonstrate that the AhR protein can function as an exogenous ligand sensor, and that these compounds appeared only recently in the human ecosystem such as polycyclic aromatic hydrocarbons (the technosphere). Thus, it can be said that the canonical pathway of AhR-Arnt may well be activated as a response to the presence of toxic compounds produced by humans, and that thanks to a process of conserved evolution, the AhR protein – belonging to a group of proteins known to be environmental sensors – is a response mechanism with the function to detoxify the organism from those “new” compounds that are foreign to nature. In this context, activation of the canonical pathway by AhR-Arnt is separated from its physiological role, in which surely many endogenous compounds perform functions less related to toxicity, and much more focused on the processes of development and differentiation of mammals, since this receptor and this group of PAS proteins also play important roles in development, a function in which AhR perhaps operates initially in the early stages of mammals, as mentioned above.

### The Non-canonical AhR Pathway

In the past 10 years, the development of research analyzing the microarrays of genes in samples of the AhR from the organs of knockout mice, and cell lines principally from the liver, and their comparison with WT mice to AhR and in normal lines of gene expression, have revealed that in the absence of ligands exogenous to the receptor exists an expression of genes for which, upon analyzing the *cis* regions of some promoters using chromatin immunoprecipitation (or CHIP), it was possible to show that they present sequences distinct from those of ARE described in the canonical pathway, which have been denominated as non-consensus response elements to xenobiotics (NC-XRE) (Peters et al., 1999; Dere et al., 2011). As a result, the non-canonical AhR pathway refers to the expression of genes which promoters even in the presence of some ligand, such as TCDD, do not contain the characteristic *cis* element of the ARE. One of these genes is the plasminogen-1 activator inhibitor (PAI-1) (Tijet et al., 2006). Later studies corroborated that treatment with TCDD suppresses hepatic regeneration, and we now understand that this is due to PAI-1 inhibition of the urokinase-type plasminogen activator that is necessary for the activation of the hepatic growth factor (Mitchell et al., 2006). The characteristic of these promoters is that they contain a repeated tetranucleotide: 5′-GGGA-3′. In the case of the PAI-1 promoter, it is the second NC-XRE that is important for the binding of AhR. One important feature is the absence of interaction with Arnt in this gene activation (Huang and Elferink, 2012). It has also been proven that suppression of hepatic regeneration is realized by stopping cell proliferation by inducing a delay in phase G_1_ by inhibiting the activity of kinase 2-dependent cyclin (CDK2) (Mitchell et al., 2006). This is dependent on the increase in the expression of kinase-dependent cyclin inhibitors such as p21 and p27. These inhibitors negatively regulate the progression of the cell cycle by controlling CDK2, CDK4 and CDK6 (Harper et al., 1995). Later, through analyses of the p21 promoter sequence, it was discovered that they contain NC-XRE *cis* regions (Jackson et al., 2015). Also, an endogenous ligand must exist that allows, in the absence of a bond to some ligand, expression of AhR-dependent p21, as was proven in the hepatic regeneration model through partial hepatectomy (Jackson et al., 2014).

Recent evidence demonstrates that the Kruppel-like factor (KLF) forms a heterodimer with the AhR receptor (Wilson et al., 2013). Structurally, the carboxy terminal of AhR is where the bHLH and PAS A domains are found that must interact with the amino terminal end of KLF6 (Wilson et al., 2013). The KLF6 factor regulates numerous cellular processes that participate in differentiation, proliferation and apoptosis (Philipsen and Suske, 1999). Alterations to the KLF6 gene are associated with various types of cancer, including astrocytomas and gliomas (Jeng and Hsu, 2003). The KLF6 factor also activates expression of p21, forming the heterodimer, which is part of the activation of the non-canonical pathway of AhR activation (Andreoli et al., 2010). The KLF6 factor induces an increase in the expression of the E-cadherin genes, transforming the growth factor and the insulin-like growth factor 1 receptor (Rubinstein et al., 2004).

## Implication of the AhR Pathway in Toxicity of Aflatoxins and Some Other Mycotoxins

It is important to understand, firstly, that the proposal for this review arose because so little has been written on this topic up to date. Secondly, we must be aware that the reports that do exist are mainly related to AFB_1_ (Ayed-Boussema et al., 2011, 2012a; Merrick et al., 2013; Mary et al., 2015), and few to *Alternaria* toxins (Pahlke et al., 2016), ochratoxin A (Ayed-Boussema et al., 2012b), fumonisin and patulin (Ayed-Boussema et al., 2011, 2012a; Mary et al., 2015). All of these studies have demonstrated the activation of members of different families of CYP during transcription (Ayed-Boussema et al., 2012b; Mary et al., 2015; Pahlke et al., 2016), enzymes with UDP-glucuronidase activity (Fleck et al., 2012; Hanioka et al., 2012), and the role played by such receptors as pregnane X (PXP), and the constitutive androstane (CAR), retinoid X (RXR), glucocorticoids (RG), and Ah receptors (Fleck et al., 2012; Hanioka et al., 2012; Mary et al., 2015; Pahlke et al., 2016). Some of these studies also evaluated the cytotoxic and genotoxic potential of these toxins (Koliopanos et al., 2002; Ayed-Boussema et al., 2012a; Fleck et al., 2012). The following describe the key results that different working groups have obtained regarding aflatoxins and other mycotoxins and their relation to the AhR.

### Activation of AhR Pathway by Aflatoxins

AFB_1_ is a powerful hepatocarcinogen (Merrick et al., 2013). Due to its planar structure and activation of transcriptional expression of the CYP1A1 in H4IIE hepatoma cells, it was suggested to be agonist for the AhR, but at the moment the direct evidence is lacking (Mary et al., 2012). Increased expression has been observed in carcinomas of the pancreas and lungs (Koliopanos et al., 2002; Lin et al., 2003). This increase in its expression is also related to an increase in the invasion of gastric cancer cells. Consequences of the binding and activation of the AhR include the induction of free radicals and ROS (Mary et al., 2012). This seems to play an important role in carcinogenesis and the progression of carcinomas. In fact, their operation functions by reducing immunovigilance of the carcinogenic process because activation of the AhR affects the regulatory function of T CD4 + lymphocytes (Pot, 2012). The activation of AFB_1_ to the AhR allows the activation of the rapid response of AhR and the activation of tyrosine kinases and the ERK and PKC1 signaling pathways (Tan et al., 2004; Schreck et al., 2012). In cultures of human hepatocytes, AFB_1_ also increased the transcriptional expression of RXR due to the increased expression of the genes of CYP2B6 and CYP3A5 cytochromes, whose expression is regulated by this receptor (Ayed-Boussema et al., 2012b).

An analysis of genes differentially-expressed by RNAseq (transcriptome) from rat livers revealed an at least twofold increase, to a total of 1026 genes. These genes participate in the AhR, NF-E2-related factor 2 (Nrf2) and glutathione (GSH) pathways, the cell cycle of the metabolism of xenobiotic compounds, the extracellular matrix and cell differentiation (Merrick et al., 2013). Observations of the H4IIE hepatocellular carcinoma lines showed activation of the AhR and of the transcription of the CYP1A1 gene (Mary et al., 2015). Finally, in HepG2 hepatocellular carcinoma cells, a significant expression of UGTs of the families 1 and 2 was observed (Hanioka et al., 2012).

There are transcription factors (TF) associated to DNA damage response. After a combination of transcriptomic analyses and prediction of binding of TF, in HepG2 cells after exposure to 5μM of AFB_1_, it was revealed that the canonical pathway is important in the response to modulate the DNA repair response by assessing the phosphorylation of H2AX histone (γH2AX), via ARNT. In the absence of ARNT, reduction in CYP1A1 expression and in γH2AX response demonstrated that canonical pathway of AhR is necessary for the DNA repair response after exposure to AFB_1_ (Smit et al., 2017).

Finally, the efflux transporter ABCG2 plays an important role in the mammary excretion of toxins in humans. AFB_1_ can interact with the ABCG2 transporter even at higher aflatoxin concentrations. This interaction produces an efflux effect of dioxin-like compounds such as PCB 126, but this function depends of AhR activation since it is inhibited by CH233191, an inhibitor of the AhR pathway. Therefore, this evidence opens new issues to research the role produced by the interaction between AFB_1_ and the close action of the AhR pathway (Manzini et al., 2017).

### Activation of AhR Pathway by *Alternaria* Toxins

*Alternaria* toxins, alternariol and its monomethyl ether are planar benzopyrones that are quite similar to B[*a*]P, compounds metabolized by CYP1A1 and 1A2 cytochromes, which generate epoxides with marked activity on DNA and protein (Schreck et al., 2012). They have both mutagenic and genotoxic effects, induce micronuclei, and show estrogenic activity (Table 1; Fleck et al., 2012; Schreck et al., 2012; Pahlke et al., 2016). They are also related to the development of esophageal cancer and are inhibitors of DNA topoisomerases (Fehr et al., 2009). Only monomethyl ether stops the cells in G2/M. Apoptosis is induced at 48 h of exposure to both compounds (Schreck et al., 2012). These two toxins are AhR activators in mouse hepatoma cells (Burkhardt et al., 2012; Schreck et al., 2012; Pahlke et al., 2016). Also altertoxin-II, at very low concentrations, induces CYP1A1, resulting in the ROS production (Pahlke et al., 2016) that can cause DNA damage, independently of the AhR, since the siRNA of the receptor does not induce CYP1A1. However, DNA damage does occur, likely due to the generation of direct DNA adducts, or the ROS induction independently of cytochrome activity (Burkhardt et al., 2012). For this reason, it is important to evaluate, as well, whether they have the capacity to induce phase II enzymes of detoxification, such as glucuronidation, because the metabolites of these mycotoxins do not cause DNA damage. This could represent a strategy for reducing the toxic properties of these mycotoxins through conjugation with glucuronic acid.

### Activation of AhR Pathway by Other Mycotoxins

So far, there have been only a few studies related to other mycotoxins, including OTA, FB_1_ and patulin as activators of AhR. In primary cultured human hepatocytes treated with subcytotoxic OTA concentrations, significant upregulation of CYP3A4, CYP2B6, and, to a lesser extent, CYP3A5 and CYP2C9 was obtained. PXR mRNA expression increased in only 1 treated liver, whereas CAR mRNA expression was not affected. OTA induced an overexpression of CYP1A1 and CYP1A2 genes accompanied by an increase in AhR mRNA expression suggesting that OTA could activate both PXR and AhR in human hepatocytes (Ayed-Boussema et al., 2012b). Also, in human kidney cells HK-2 OTA activated both AhR and PXR by induction of the CYP1A1, CYP1A2, and CYP3A4 genes. The mRNA expression of phase II enzymes such as heme oxygenase-1, nicotinamide adenine dinucleotide phosphate-quinone oxidoreductase 1, and glutamate cysteine ligase catalytic subunit were upregulated by the activation of Nrf2. Nrf2 activation is directly induced by OTA-activated AhR and PXR, or indirectly by the adaptive response to ROS generated through metabolic processes between AhR, PXR-induced CYP enzymes, and OTA. Taken together, these studies indicate that OTA induces phase I and II enzymes through the activation of AhR, PXR, and Nrf2 signaling pathways, which may lead to cell injury (Lee et al., 2018).

Induction of CYP enzymes by FB_1_ has been poorly investigated so far; but an increase of CYP1A activity in the liver of Wistar rats exposed to FB_1_ has been confirmed (Martínez-Larrañaga et al., 1996). Recent study demonstrated that FB_1_ applied alone caused a small increase in CYP1A activity and *cyp1A* gene transcription in H4IIE and rat spleen mononuclear cells, but to a lesser extent and duration than single AFB_1_. However, *cyp1A* gene expression by FB_1_ applied alone did not involve Ahr activation. On the other hand, a two-toxin mixture induced an overexpression of *cyp1A* and *ahr* genes in spleen cells indicating the interaction between both toxins in AhR pathway activation and subsequently in carcinogenesis (Mary et al., 2015).

In primary human hepatocytes, PAT induced an upregulation of PXR and CYP2B6, 3A5, 2C9, and 3A4 genes accompanied by AhR and CYP1A1 and 1A2 mRNA expression. Since this is the only study available in literature related to AhR activation by PAT, further studies should determine whether this mechanism could modulate PAT toxicity (Ayed-Boussema et al., 2012a).

## Conclusion and Perspectives

‐ Due to their toxic properties, aflatoxins and other mycotoxins are AhR activators and are also mutagenic and carcinogenic.
‐ As lipophilic compounds, they can easily cross cells biological membranes that are a part of the entrance pathway; for example, the intestinal wall. For this reason, they constitute as pro-carcinogenic molecules.
‐ Their activation as carcinogenic metabolites has centered on modification by mono-oxygenases, represented primarily by CYP1A1, that are expressed abundantly in the liver. Furthermore, the CYP1A1 promoter has 8 XRE-elements which are used by the AhR activation.
‐ The gene expression increase and the activity of such cytochromes such as CYP1A1 are important factors due to their activity, triggered by exposure to aflatoxins can go on for weeks or months, meaning that the carcinogenic process can be sustained due to the consequences of this activity.
‐ Aflatoxin metabolites are mutagenic and some produce adducts on the nitrogenous bases that can cause base changes and modifications in the methylation states of gene promoters and thereby establish a modified expression pattern. This area has not been yet fully explored.
‐ These metabolites also induce an increase in ROS that can cause damage to DNA, lipids or other proteins, and thereby initiate the process of carcinogenesis.
‐ Mutations can be produced in genes with important functions but they are also targets for mutagen modification such as p53 and K-Ras, among others.
‐ The increase in mRNA expression of the AhR is significant due to significant expression of this gene in liver and adipose tissue, with the latter potentially serving as a reservoir for lipophilic compounds like aflatoxins. It is therefore important to mention that in the AhR promoter there are several important *cis* elements which have an important function in interactions with *trans* factors. Some *cis* elements are: HNF (binding of the hepatic nuclear factor); DLX3 (regulatory placenta-specific); BRN2-like (binding of factors participating in neural development and adult stage in mammals, also performing differentiation of sensitive and motor neurons); STAT6 (responding to cytokines such as IL-4 and TGF-β1); Tcf/Lef (Wnt via/neurodevelopment). Binding sites to potential nuclear receptors to the progesterone receptor, androgen receptors, glucocorticoid receptors, proliferation-activated peroxisome receptors, the farsenoid X receptor and the vitamin D receptor could explain the biological effects of these toxins. These *cis* elements partly enable the comprehension of the toxic effects caused by the activation of AhR, but this is a less studied area.
‐ Up to date, the AhR activation has been limited to groups of genes such as the cytochromes and UDP-glucuronidases. However, we know that AhR activation can participate in inflammatory responses, cell proliferation and differentiation, and the loss of cell adhesion. Thus, the implications of AhR activation by aflatoxins may be more serious than thought thus far.
‐ Certain polymorphisms can predispose tissues to cancer development, when they are exposed to aflatoxins. This field needs to be studied broadly since we know that at least some polymorphisms of enzymes like GCTP1, AKR, EPHX1, and EERC2 may participate in cancer development.
‐ The pathways that depend on the AhR activation have been explored, but there must be others, such as the non-canonical receptor pathway that participates in the toxic and carcinogenic effects by aflatoxins. At this point it is important to emphasize that AhR protein heterodimerizes with KLF6 and this complex can activate CYP1A1. Finally, KLF6 can regulate differentiation, proliferation and apoptosis.
‐ The study of the aflatoxins effects thus represents an area of opportunity in which diverse specialists, including researchers in omics, must work together in efforts to integrate the data that make their field of study so complex.

## Author Contributions

FA-H, RV-Q, and MS contributed mostly in final shaping and improving the manuscript, while the other authors also contributed in writing but also in designing the tables, figures, and correcting the manuscript with English and reference editing. All authors have contributed to the idea and form of the manuscript.

## Conflict of Interest

The authors declare that the research was conducted in the absence of any commercial or financial relationships that could be construed as a potential conflict of interest.
